# Trajectories of relapse in randomized placebo-controlled trials of treatment discontinuation in major depressive disorder: an individual patient level data meta-analysis

**DOI:** 10.1016/S2215-0366(17)30038-X

**Published:** 2017-02-09

**Authors:** Ralitza Gueorguieva, Adam M. Chekroud, John H. Krystal

**Affiliations:** 1 Department of Biostatistics, School of Public Health, Yale University School of Medicine, 60 College St, New Haven, CT; 2 Department of Psychology, Yale University, 2 Hillhouse Avenue, New Haven, CT; 3 Spring Health, New York City, NY; 4 Centre for Outcomes Research and Evaluation, Yale-New Haven Hospital, CT; 5 Department of Psychiatry, Yale University School of Medicine, Suite 901, 300 George St., New Haven, CT; 6 VA National Center for PTSD, VA Connecticut Healthcare System, Psychiatry (151D) Building 35, West Haven, CT

## Abstract

**Background:**

Understanding patterns of relapse in antidepressant treatment responders can inform strategies for preventing relapse.

**Methods:**

We re-analyzed individual-patient data from four double-blind discontinuation clinical trials of duloxetine or fluoxetine vs. placebo in major depression (N=1462). Trajectories of depression severity (Hamilton Depression Rating Scale scores) were identified in the entire sample, and separately in arms where antidepressant had been continued or discontinued. Predictors of trajectory membership were assessed.

**Findings:**

We identified similar “relapse” trajectories and two trajectories of stable depression scores in the normal range on active medication and on placebo. Active treatment (OR=0.47, 95% CI: (0.37, 0.61)) significantly lowered the odds of membership in the “relapse” trajectory whereas female sex (OR=1.56, 95% CI: (1.23, 2.06)), shorter length of time with clinical response (OR=1.10, 95% CI: (1.06, 1.15)) and higher Clinical Global Impressions score at baseline (OR=1.28, 95% CI: (1.01, 1.62)) increased the odds. Overall, the protective effect of antidepressant medication relative to placebo on the risk of being classified as a relapser was about 13% (46% vs. 33%).

**Interpretation:**

The existence of similar relapse trajectories on active medication and on placebo suggests that there is no specific relapse signature associated with antidepressant discontinuation. Furthermore, continued treatment offers only a modest protection against relapse. These data highlight the need for incorporating treatment strategies that prevent relapse as part of the treatment of depression.

## Introduction

Major depressive disorder (MDD) typically follows a recurrent course ^1^. On average, individuals with a history of depression who respond to treatment have a 30-50% chance of relapse within one year^2^ and they will have five to nine separate episodes in their lifetime^3^. The risk of relapse is reduced by maintenance interventions including pharmacotherapy^4^ or psychosocial treatments^5^. Clinical trials evaluating relapse prevention approaches generally attempt to reduce the proportion of patients who relapse within a pre-determined time period (i.e., 4-6 months), where relapse is defined as surpassing a cut-point on an aggregate severity scale (i.e., Hamilton Depression Scale (HAMD) score ≥ 14). However, it has been noted that this transformation of continuous data to categorical data (i.e. “relapse” or “non-relapse”) can amplify small mean differences, which may obscure the evaluation of the clinical importance of therapeutic interventions^6-8^.

With this in mind, there has been a growing interest in using trajectory-based approaches to analyze clinical trial data, particularly in trials attempting to produce an initial remission of depression symptoms^6-9^. Trajectory-based models (e.g. latent class models^10^, and growth mixture models^11^) capture heterogeneity in the development of clinical outcomes during an intervention, and this more sensitive approach can result in trial outcomes that differ from traditional endpoint measures^8,12^. Additionally, they enable the identification of distinct classes of time-dependent treatment responses and the evaluation of treatment effects upon trajectory membership. This approach has identified distinct classes of antidepressant response trajectory, including rapid or gradual improvement^8^, transient improvement followed by symptom worsening^9^, or “non-responders” who do more poorly on medication than placebo^6^.

Far less is known, however, about trajectories of relapse for patients who initially responded to treatment and who either continued or discontinued medication treatment. To study this issue, we applied growth mixture modeling to identify distinct trajectories of HAMD scores using individual patient level data pooled from four randomized double-blind placebo-controlled discontinuation trials of duloxetine and fluoxetine. In particular, we explored whether similar or different trajectory classes exist for patients who continued active treatment or who discontinued active treatment, and tested whether there were clinical predictors of trajectory class membership. Applied in this context, these methods provided new insights into the stability of clinical response and the trajectory of relapse to depression.

## Methods

### Sample

We analyzed data from four randomized, multicenter, double-blind, placebo-controlled discontinuation clinical trials of duloxetine and fluoxetine for MDD conducted by Eli Lilly prior to 2012. Table 1 describes the studies, arms, sample sizes and duration. Four different protocols were followed (protocol identifiers HCIZ, HCEX, HMBC and HMDI). All studies incorporated an open-label acute treatment phase with either duloxetine or fluoxetine and a double-blind discontinuation phase where patients continued their medication or received placebo. Two of the studies (HCIZ and HMBC) had an optional rescue phase that was not included in this analysis. Flow-charts of the protocols and a summary of inclusion/exclusion criteria are included in the supplemental materials (Figures S1-S4). Results from time-to-relapse analyses are published elsewhere^13-16^ (Table S1). We modelled trajectories of relapse up to 26 weeks during double-blind treatment. Data were aligned so that the following time points were used: weeks 0, 2, 4, 10, 16, 22 and 26 (Table S2).

### Statistical analysis methods

The outcome variable was total score on the 17-item HAMD scale. We used growth mixture modeling^11^ to identify distinct trajectories of HAMD scores during treatment discontinuation. We first fitted models to the entire sample and then fitted separate models to the placebo and active arms separately. The latter analyses were used to evaluate whether different classes would emerge for subjects in the active arms and in the placebo arms. We considered linear and quadratic trends over time and up to four trajectory classes. The selection of the best model was based on the Schwartz-Bayesian Information Criterion (BIC) and on the Lo-Mendell-Rubin likelihood ratio test (LMR)^17^. The LMR test compares the fit of a model with two or more classes to a model with one fewer class in order to identify the optimal number of classes. We only considered models where the smallest class had at least 5% of the total subjects so that the resulting model would be meaningful clinically and stable numerically. Classification confidence was assessed using the entropy value ranging between 0 and 1, with higher values corresponding to higher confidence in latent class assignments.

To evaluate whether the resulting trajectories were consistent across the different trials, we also performed separate trajectory analyses by protocol. In this secondary analysis, treatment was included as a predictor in the entire sample and by protocol in order to assess whether there were significant treatment effects on trajectory membership.

Trajectories during discontinuation were classified as “relapse” vs. “non-relapse”. We assessed the association between the most likely trajectory classification of the individuals and a simple categorical indicator of relapse (HAMD ≥ 14 at the last available assessment point) using Fisher's exact tests and conditional probabilities.

Weighted logistic regression was performed to assess the effects of treatment (during open-label and during the double-blind discontinuation phase), length of time with clinical response and subject characteristics on membership in a particular class. Study protocol was not included because it was confounded with treatment and is not useful as a predictor outside of these data. Interactions between treatment and the covariates were considered in order to assess potential moderating effects, but were dropped from the final model because they were not statistically significant. We calculated the length of time with clinical response as the number of weeks between randomization and time when HAMD score fell below 10 during the open-label phase. Other characteristics included sex, age, age of onset of first episode, number of previous episodes (0, 1 or 2, 3 or 4, 5 or more, missing) and CGI-severity score at randomization to discontinuation treatment. The weights were the posterior probabilities of membership in the assigned class. The association of each predictor with trajectory membership was also tested one at a time using t-tests, chi-square tests or Fisher's exact tests. Odds ratios and 95% confidence intervals were used to estimate effect sizes for the different predictors.

We also performed weighted logistic regression with the same predictors but with the simple clinical definition of relapse (HAMD score of 14 or higher) in order to assess the robustness of predictors of relapse to the definition of relapse. Identification of latent trajectory classes was performed using MPlus^9^ and all other analyses were conducted in SAS.

## Results

In the entire sample, as well as in the samples receiving active medication and placebo during the discontinuation phase, we selected the models with three trajectory classes (Table 2). The models with three trajectory classes fit better according to both the BIC and the LMR statistic than the models with fewer classes in all analyses. Models with more than three classes could not be estimated reliably (i.e., the best-likelihood value could not replicated, the estimated variance-covariance matrix in one or more classes was not positive definite or the number of subjects per class was less than 5%) and hence are not presented. Separate analyses of the studies also identified three trajectory classes with similar shapes over time (Figures S5-S8).

**Figure 1:**
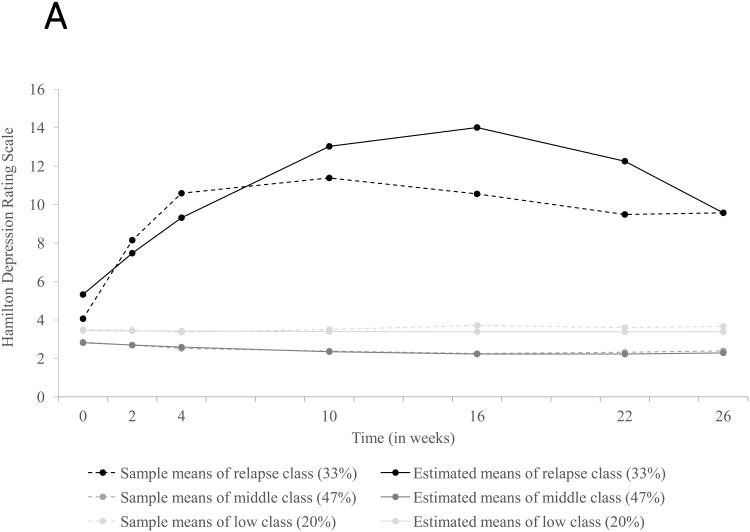

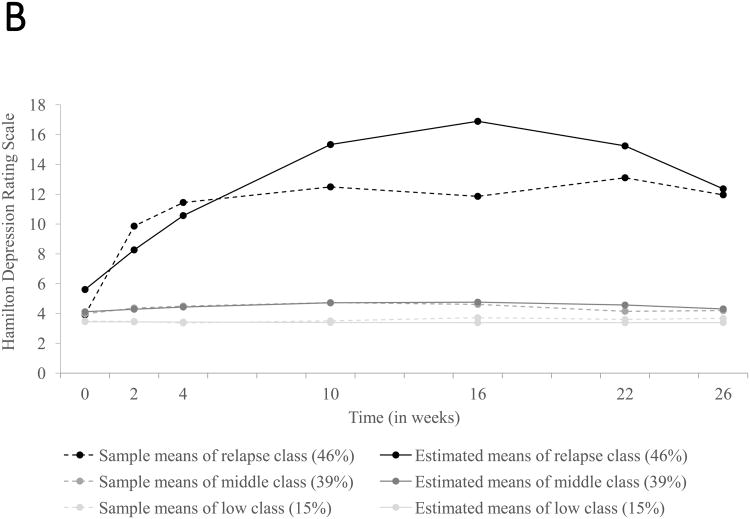
**A:**Trajectories of HAMD scores in the active arms. **B:**Trajectories of HAMD scores in the placebo arms.

Figure 1 shows the estimated and sample means for the three trajectory classes over time for the samples on active medication and on placebo. The trajectory classes in the entire sample were very similar. The two classes on the bottom of both figure panels show flat HAMD trajectories over time well below the symptomatic range (HAMD scores below 5) with slightly more separation between the two classes on active medication than on placebo. We refer to these classes as “non-relapse” classes. They differ slightly in their mean scores but also there are more fluctuations in scores over time in the higher non-relapse class than in the lower relapse class (Figure S9). The third class shows rapidly increasing HAMD scores (to above 10) during the discontinuation phase with slightly higher scores on placebo but the shape of these trajectories in subjects on active medication and on placebo are very similar. We refer to this class as the “relapse” class. HAMD data on subjects after they meet clinical criteria for relapse in the studies are not reported, as they entered “rescue” treatment. As a result, the sample mean trajectories for the “relapse” class are somewhat below the estimated mean trajectories for the same class in all analyses. However, it is unlikely that missing data influences the reported findings substantially (specifically the separation of relapse vs. non-relapse trajectories), as growth mixture models provide valid results under the assumption that data are randomly missing and trajectory up until relapse predicts the loss of data. Sensitivity analysis using pattern-mixture models^9^ investigating stability of latent classes under missing not at random assumptions failed to identify stable trajectory classes.

The estimated probability of membership in the relapse class is 45.8% on placebo and 33.1% on active medication. Almost all (944 out of 947, 99.7%) of patients who were classified as non-relapsers based on the trajectory analysis with trajectories 1 and 2 combined, did not relapse according to the simpler clinical relapse criterion of a HAMD score of 14 or higher. The percentages were almost the same when calculated by treatment group: 99.5% (660 out of 663) on active medication and 100% (all 284 individuals) on placebo. More than two thirds (365 out of 515, 70.9%) of the individuals most likely to follow the “relapse” trajectory relapsed according to the simpler clinical definition of relapse, with a higher rate for individuals on placebo (75.2%, 164 out of 218) than for individuals on active medication (67.7%, 201 out of 297). Thus almost a third of individuals on active medication (32.3%, 96 out of 297) and about a quarter of the subjects on placebo (24.8%, 54 out of 218) who were following the relapse trajectory did not meet traditional clinical definitions of relapse. Those individuals had on average lower mean depression scores than those who relapsed according to both definitions (Figure S10).

Univariate associations between the trajectory classes identified in the joint analysis of active and placebo arms (grouped as “relapse” vs. “non-relapse”) and treatments, study protocol and covariates are provided in Table 3. When adjusting for uncertainty in trajectory membership and other covariates, active treatment during discontinuation halved the odds of following the relapse trajectory (OR=0.47, 95% CI: (0.37, 0.61)) while female gender (OR=1.56, 95% CI: (1.23, 2.06)), shorter length of time with clinical response by 1 week (OR=1.10, 95% CI: (1.06, 1.15)) and higher CGI severity by 1 (OR=1.28, 95% CI: (1.01, 1.62)) significantly increased the odds of following the relapse trajectory. Accuracy in predicting whether a patient would be in the relapse trajectory or not was reasonable (AUC = 66%, Figure S11), especially given the small number of baseline predictors available for analysis. The results from the weighted logistic regression with simple HAMD remission definition (HAMD score of 14 or more) were very similar (see Table 4).

## Discussion

The protective effect of antidepressant medications on depressive relapse is a cornerstone of psychiatry and one that has yielded the recommendation that patients with recurrent depression remain on antidepressant treatment for the remainder of their lives^18,19^. This study analyzed data from four clinical trials aimed at evaluating the risk for relapse when patients who had responded to treatment with fluoxetine or duloxetine were blindly maintained on their medication or switched to placebo. The principal finding was that trajectory-based analyses revealed the same three response trajectories in patients who stayed on their medications or were switched to placebo. This suggests that there is no specific relapse signature associated with antidepressant discontinuation.

The first two trajectories we identified constituted the majority of patients, showed sustained clinical response over 26 weeks, and respected traditional symptom thresholds for remission extremely closely. Individuals in the lowest severity trajectory had low scores and low variability of scores from visit to visit. The middle severity trajectory showed more score instability and slightly higher depression scores (still in the subclinical range). Since both groups had good outcomes we did not explore differences in characteristics between them in this study. Our main focus was on the relapse trajectory of increasing depression scores, in which about 46% of patients treated with placebo and 33% of patients treated with active medication were categorized.

Within the relapse trajectory, over 70% of patients also met symptomatic criteria for relapse but close to 30% did not. Trajectories of relapse may be informative even if clinical relapse criteria are not met, since prediction of trajectory membership could occur early on and since clinical relapse criteria are somewhat arbitrary. Patients who follow a relapse trajectory but do not meet criteria may have effectively relapsed nonetheless or may be at an increased risk of relapse in the future. Although on average this group had lower depression scores than the group of individuals identified as relapsers by both the trajectory and clinical criteria in this study, the absence of longer term follow-up data precluded us from comparing their longer term outcomes. Future studies are needed to evaluate this question.

The high rate of relapse suggests that short-term antidepressant response is not very stable and the similarity of relapse trajectories on active medication and on placebo indicates that the temporal dynamic of mood regulation is not altered by SRI treatment. Approximately a third of the patients in this study followed the relapse trajectory, which is consistent with the findings of other studies^5,20^. Nevertheless, this should not be interpreted as evidence to downplay the benefit of SRIs during the initial episode. Furthermore, SRIs appeared to protect against the natural tendency to relapse during maintenance, i.e. they make patients more resilient. The likelihood of relapse was also related to length of time with clinical response ^21^, level of residual symptoms^21^, and was greater for women than men^22^. One possible explanation for these observations suggests that the efficacy of SRI antidepressants continues to be consolidated long after initial symptom reductions have occurred. At the moment, we do not understand this consolidation process, although structural neurobiological changes might be one part of it. It is striking that short-term antidepressant response is not particularly stable but that demonstration of long-term antidepressant efficacy is not required for approval by the U.S. Food and Drug Administration (FDA). One wonders whether it would be valuable to expect evidence of long-term efficacy when new antidepressants are approved by the FDA. This concern is somewhat reduced by evidence that early antidepressant response is a relatively strong predictor of later response ^23^.

The current study suggests that SRI antidepressants have only a modest protective effect against relapse relative to placebo, as reflected in an approximately 13% difference in the likelihood of being in the relapse trajectory. This suggests that SRI treatment by itself leaves many patients at risk and specific strategies for preventing relapse should be more widely implemented in depression treatment. In the future, one hopes that this research can be extended by identifying moderators of treatment effects. That is, to ultimately identify the type and intensity of treatment that would maximize the probability of a desired outcome for that specific patient. Until then, non-specific predictors are still useful for setting prior expectations about clinically relevant outcomes, e.g. relapse or initial treatment response ^23,24^. The application of machine learning methods to a much broader array of predictive markers has proven successful in other areas of psychiatry, particularly predicting treatment outcomes^23,25^

In light of the current data, it may be important to develop new and more cost effective psychosocial treatments to reduce depression relapse in order to ensure widespread implementation. Interpersonal psychotherapy (IPT), for example, decreases depression relapse^26^, but it does not appear to reduce treatment costs^19^ and it is less effective in preventing relapse in patients who did not respond to IPT alone, but did respond when pharmacotherapy was added^27^. The development of more effective pharmacologic relapse prevention strategies might also improve outcomes for patients with unipolar depression. Lithium, for example, reduces relapse for mood disorders overall, but does not show clear efficacy in preventing relapse for patients with unipolar depression^28^. More broadly, the strategic integration of psychotherapy and medication for relapse prevention is a critical issue for patients and for the field^29^, especially in avoiding common clinical problems associated with long-term antidepressant treatment^30^.

One potential limitation of three of the four protocols is the absence of rigorous measures of antidepressant withdrawal symptoms. Withdrawal symptoms for duloxetine and fluoxetine most commonly include dizziness, nausea, headache, but may also include worsening of anxiety or depression^31^. Some withdrawal symptoms may persist for several weeks after antidepressant discontinuation^32^. Fluoxetine has among the longest half-life from among the SRI medications, i.e., up to 3 days which may protect patients from some withdrawal symptoms, while duloxetine has a much shorter elimination half-life (approximately 10 hours) and would therefore be viewed as having more potential to produce withdrawal symptoms. However, we did not observe a trajectory consistent with the emergence and abatement of withdrawal symptoms on placebo. This may be at least partially attributable to the fact that some studies tapered off antidepressant medications over several weeks, which could have further limited the impact of post-discontinuation symptoms on the results. Indeed, this makes the results more reflective of clinical practice. These findings reduce our concern that the results were substantially contaminated by the appearance of antidepressant withdrawal symptoms.

This study has other limitations. Firstly, there is a potential for expectancy bias since the analyses are based on discontinuation studies which may carry some greater expectation of relapse. Secondly, we did not have data available about these patients to adjust for intercurrent major life stress, which contributes to relapse while treated with medications^33^. Thirdly, patients exited the study upon relapse (to move into a rescue phase), which is likely why predicted trajectories for the relapse class are slightly inflated relative to the observed mean trajectories^9^. Fourthly, quadratic models do not capture the curvature in the relapse trajectory very well. More complicated models such as latent basis growth mixture models could provide better fit^34^. Lastly, we had a limited number of predictors to relate to relapse trajectories thus there might be much stronger predictors that might be useful in reducing the probability of relapse^35^. In particular, other biological factors may contribute to the association between elevated CGI, gender and depression. Important future work will be to identify additional predictors of relapse, and other clinical features associated with these relapse trajectories in line with the NIMH RDoC framework, as well as eventually advancing our understanding of neurobiological mechanisms related to relapse.

## Conclusion

The similarity of trajectories on active medication and on placebo suggests that there is no specific relapse signature associated with antidepressant discontinuation. The current study supports the continued prescription of SRI antidepressants to protect against relapse of depression. However, it suggests that this protective effect is less than one might have expected. Patients and providers should be prepared for the possibility that as many as one of three patients who initially respond to an antidepressant will worsen over the subsequent six months, which justifies a more widespread effort at preventing relapse in patients with unipolar major depression.

## Supplementary Material

supplement

## Figures and Tables

**Table 1:** Discontinuation clinical trials for patients with major depression.

Protocol	Duration of acute phase open label treatment	Treatment during acute phase	Duration of double-blind discontinuation phase	Total Sample Size	Arms and sample sizes per arm during discontinuation phase			
HCIZ	13 weeks	Fluox 20mg/day	25 weeks	501	Fluox 20mg/day 189	Fluox 90mg/week** 190		Placebo 122
HCEX	12 weeks	Fluox 20mg/day	14-50 weeks (see arms)	395	Fluox 20mg QD 50 weeks 102	Fluox 20mg QD 38 weeks 100	Fluox 20mg QD 14 weeks 97	Placebo 96
HMBC	12 weeks	Dulox 60mg QD	26 weeks	288	Dulox 60mg QD 136			Placebo 142
HMDI	10 + 24 weeks*	Dulox 60-120mg QD	52 weeks	278	Dulox 60mg QD 64	Dulox 90mg QD 45	Dulox 120mg QD 37	Placebo 142

* Patients underwent up to 10 weeks open label acute therapy phase during which dose was optionally increased to 90 or 120mg QD in the case of non-responses. Patients who met response criteria during week 4 to 10 of the acute open-label treatment were moved directly to the 24 week open-label continuation therapy phase.

** Enteric coated fluoxetine 90mg once a week.

**Table 2:** Results from model selection for the entire sample and for the subsamples of subjects on active medication and on placebo.

	Likelihood	BIC	Entropy	Lo-Mendell-Rubin test	Proportion of Individuals In Class*			
				2log-lik. P-value	1	2	3	4
**All arms**								
1-class model	-22782.46	45695.13			1.00	--	--	--
2-class model	-19695.07	39499.45	0.82	327.46 <.0001	0.56	0.44	--	--
3-class model	-19363.41	38879.85	0.78	672.5 <0.0001	0.44	0.37	0.19	--
**Only placebo arms**								
1-class model	-7024.31	14092.15			1.00	--	--	--
2-class model	-6535.92	13114.47	0.83	954.84 <.0001	0.51	0.49	--	--
3-class model	-6445.19	13020.96	0.79	177.40 0.05	0.46	0.39	0.15	--
**Only active arms**								
1-class model	-14176.91	28401.88			1.00	--	--	--
2-class model	-12992.38	26101.49	0.77	1795.52 <0.0001	0.54	0.46	--	--
3-class model	-12883.43	25911.06	0.78	486.51 0.0002	0.47	0.33	0.20	--

* Classes are ordered by class size and do not necessarily correspond to one another across samples. Four-class models could not be reliably estimated.

**Table 3:** “Trajectory relapsers” and “trajectory non-relapsers” by treatment, study and baseline characteristics.

	“Relapsers” (N=515, Row %=35.2%)	“Non-relapsers” (N=947, Row %=64.8%)	Total (N=1462)
**Drug during open label treatment**			.004
Duloxetine	174 (30.7%)	392 (69.3%)	
Fluoxetine	341 (38.1%)	555 (61.9%)	
**Protocol**			<.0001
HCIZ	214 (42.7%)	150 (57.3%)	
HCEX	127 (32.2%)	86 (67.9%)	
HMBC	120 (43.2%)	90 (56.8%)	
HMDI	54 (18.8%)	155 (81.3%)	
**Drug during discontinuation**			<.0001
Active	297 (30.9%)	663 (69.1%)	
Placebo	218 (43.4%)	284 (56.6%)	
**Gender**			0.0005
Female	390 (38.1%)	634 (61.9%)	
Male	125 (28.5%)	313 (71.5%)	
**Number of previous episodes**			0.0002
0	39 (32.2%)	82 (67.8%)	
1-2	136 (37.5%)	227 (62.5%)	
3-4	111 (27.1%)	298 (72.9%)	
5 or more	120 (37.3%)	202 (62.7%)	
missing	109 (44.1%)	361 (55.9%)	
**Baseline CGI**			0.09
1	311 (33.3%)	623 (66.70%)	
2	202 (38.9%)	318 (61.15%)	
3	2 (28.6%)	5 (71.43%)	
**Quantitative predictors**	**Mean (SD)**	**Mean (SD)**	
**Length of time with clinical response**	6.50 (3.17)	7.58 (3.36)	<.0001
**Age**	42.18 (11.27)	43.53 (12.08)	0.04
**Age of onset**	21.48 (14.94)	22.99 (17.01)	0.08

**Table 4:** Odds ratios and 95% confidence intervals for the effects of predictors on relapse trajectory membership and relapse defined as HAMD score of 14 or more.

	Relapse trajectory	HAMD score of 14 or more
**Categorical predictors**		
**Drug during discontinuation**		
Active vs. Placebo	0.47 (0.37, 0.61)	0.45 (0.35, 0.59)
**Drug during open label treatment**		
Duloxetine vs. Fluoxetine	0.88 (0.64, 1.21)	0.83 (0.59, 1.18)
**Gender**		
Female vs. Male	1.59 (1.23, 2.06)	1.71 (1.28, 2.29)
**Number of previous episodes**		
1-2 vs. 0	1.28 (0.78, 2.10)	1.33 (0.78, 2.28)
3-4 vs. 0	1.00 (0.60, 1.67)	1.06 (0.61, 1.87)
5 or more vs. 0	1.40 (0.86, 2.29)	1.34 (0.78, 2.30)
Missing vs. 0	1.86 (1.11, 3.12)	1.58 (0.90, 2.77)
**Baseline CGI**		
**2 or 3 vs. 1**	1.30 (1.02, 1.66)	1.34 (1.03, 1.74)
**Quantitative predictors**		
**Shorter vs. longer duration of response by one week**	1.10 (1.06, 1.15)	1.12 (1.07, 1.17)
**Older vs. younger age by 1 year**	1.00 (0.99, 1.00)	1.00 (0.99, 1.01)
**Older vs. younger age of onset by 1 year**	1.00 (0.99, 1.01)	1.00 (0.99, 1.01)

## Research in Context

### Evidence before this study

Discontinuation trials, in which patients are randomized to stay on an effective treatment or to blindly discontinue this treatment, provide unique insights into the stability of antidepressant response. We searched PubMed from inception to Jan 10, 2017 with the terms (“depression” OR “major depressive disorder”) AND “discontinuation” AND “trial” in any field, with no language restrictions. We retrieved and scanned 833 articles, then focused on the 346 articles in which (“depression” OR “major depressive disorder”) was in the title. All articles that we deemed not to be relevant on the basis of their titles were excluded. Abstracts of the remaining articles were reviewed to identify potentially relevant articles, and, on the basis of this selection, we read full-text articles.

Epidemiological and clinical evidence indicates that major depressive disorder (MDD) typically follows a recurrent course, with one-third to one-half of patients relapsing within one year of discontinuation. Clinical trials that examine relapse prevention approaches generally attempt to reduce the proportion of patients who relapse within a pre-determined time period (i.e., 4-6 months), where relapse is defined as surpassing a cut-point on an aggregate severity scale (i.e., Hamilton Depression Scale (HAMD) score ≥ 14). However, it has been noted that this transformation of continuous data to categorical data (i.e. “relapse” or “non-relapse”) can amplify small mean differences, which may obscure the evaluation of the clinical importance of therapeutic interventions.

Trajectory-based models (i.e. latent class based approaches) provide a data-driven method to identify distinct classes of time-dependent treatment responses, and for evaluating the effect of treatment upon trajectory membership. This approach has identified distinct classes of antidepressant response trajectories, including rapid or gradual improvement, transient improvement followed by symptom worsening, or “non-responders” who do more poorly on medication than placebo. However, to our knowledge this approach has not been used to identify distinct trajectories during discontinuation clinical trials.

### Added value of this study

The objective of the study was to estimate trajectories of relapse in responders to antidepressant treatment for major depression who remained on active treatment or were switched to placebo. We analyzed individual-patient level data from four double-blind randomized placebo controlled discontinuation clinical trials of fluoxetine or duloxetine. We identified the same three patterns over time across multiple double-blind treatment continuation or discontinuation studies, i.e. we found no evidence that there are distinct trajectories of relapse during discontinuation on active medication and on placebo. Compared to a simple clinical definition of relapse (depression severity of 14 points or above on the HAMD), the trajectory approach identified individuals likely to follow a relapse trajectory who do not meet simpler criteria for relapse.

The protective effect of antidepressant continuation treatment was modest, with only about 13% difference between the estimated proportion of individuals following a relapse trajectory on active medication (33%) vs. placebo (46%). In addition to treatment, female sex, shorter time with clinical response and poorer Clinical Global Impression score at baseline all predicted that a patient would be in the “relapse” trajectory. In summary, this study identified trajectories of relapse, predictors of patterns of increasing HAMD scores, and provided evidence for a statistically significant but a modest protective effect of antidepressant treatment.

### Implications of all the available evidence

#### Clinical implications

It is important for providers and consumers of depression treatment to understand the actual benefits of antidepressant treatments. For example, the STAR*D study suggested that one in three patients will have a full clinical response to their initial antidepressant. The current study suggests that about one-third of clinical responders will relapse even if they continue with the medication that produced their initial clinical response, while nearly 50% of patients will experience a return of depression symptoms if they stop their medications. However, the protective effect of continued medication is much smaller, only 13%, than one might have expected or hoped for. These findings suggest that strategies for reducing or forestalling the return of depression symptoms need to be developed and widely implemented in depression treatment.

#### Methodological implications

Latent class techniques can be used for data-driven identification of patterns of depression severity during acute and discontinuation treatment. Our results confirm previous research using simple dichotomous definitions of response that the majority of patients maintain clinical response regardless of whether they continue active medication. Future studies to identify predictors of trajectories of relapse are indicated.
