# Supplementary material for: Trajectories of relapse in randomized placebo-controlled trials of treatment discontinuation in major depressive disorder: an individual patient level data meta-analysis
Source: Lancet Psychiatry. Author manuscript; Available in PMC 2018 Mar 1. (PMC5340978; doi:10.1016/S2215-0366(17)30038-X)
Supplement: supplement [file NIHMS852766-supplement.pdf]

Table S1: Original publications, location, inclusion and exclusion criteria of the four studies used to identify trajectories of relapse during discontinuation of antidepressant treatment for major depression.

Protocol	HCIZ	HCEX	HMBC	HMDI
Study	Schmidt ME, Fava M, Robinson JM, Judge R. **The efficacy and safety of a new enteric-coated formulation of fluoxetine given once weekly during the continuation treatment of major depressive disorder.** J Clin Psychiatry 2000; 61(11):851-7.	Reimherr FW, Amsterdam JD, Quitkin FM, Rosenbaum JF, Fava M, Zajecka J, Beasley C, Michelson D, Roback P, Sundell K. **Optimal Length of Continuation Therapy in Depression: A Prospective Assessment During Long Term Fluoxetine Treatment**. Am J Psychiatry Sept 1998; 155(9):1247-1253.	Perahia DG, Gilaberte I, Wang F, Wiltse CG, Huckins SA, Clemens JW, Montgomery SA, Montejo AL, Detke MJ. **Duloxetine in the prevention of relapse of major depressive disorder: Double-blind placebo-controlled study.** Br J Psychiatry. 2006; 188: 346-353.	Perahia DG, Maina G, Thase ME, Spann ME, Wang F, Walker DJ, Detke MJ. **Duloxetine in the prevention of depressive recurrences: A randomized, double-blind, placebo-controlled trial.** J Clin Psychiatry. 2009; 70(5):706-716.
Location	42 study centers in the United States	Five outpatient clinics in the United States	France, Italy, Spain and the USA.	France, Germany, Italy, Russia, Sweden and the USA.
Inclusion criteria	18-80 years;<br>male or female outpatients;<br>met DSM-IV criteria for nonpsychotic major depression with a current episode duration of at least 4 weeks and HAMD-17 score of at least 18 and a Clinical Global Impressions-Severity of Illness (CGI-S) scale score of at least 4;<br>responders to 13 weeks of 20mg daily fluoxetine treatment (i.e. no longer meeting criteria for major depressive episode and had a modified HAMD-17 score of 9 or lower and CGI-S core of 2 or lower for the last 2 visit of the open-label treatment period);<br>provided written informed consent.	18-65 years;<br>male of female outpatients;<br>met DSM-III-R criteria for major depression with a duration of at least 1 month;<br>modified 17-item Hamilton scale (HAMD-17) score of at least 16 prior to the open-label phase;<br>remitted (no longer meeting DSM-III-R criteria after 12-14 week open-label acute therapy phase of fluoxetine 20mg/day and HAMD-17 score less than 7 for 3 consecutive weeks);<br>provided written informed consent.	At least 18 years old;<br>male or female;<br>met DSM-IV criteria for MDD without psychotic features;<br>HAMD-17 score of 18 or more and CGI-S score of 4 or more prior to open-label duloxetine phase;<br>at least one other major depressive episode before the episode that was being experienced at the time of study entry;<br>response during the open-label phase (HAMD-17 score of 9 or less; CGI-score of 2 or less and no longer meeting DSM-IV criteria for MDD in weeks 10 and 12 of the acute phase);<br>provided written informed consent.	At least 18 years old;<br>male or female;<br>met DSM-IV (confirmed by MINI) criteria for recurrent MDD;<br>HAMD-17 of 18 or more and CGI-S score of 4 or more;<br>at least 3 episodes of depression including the presenting episode within the past 5 years;<br>in remission between the three episodes of depression; stable and off antidepressant medication for at least 2 months prior to the onset of the presenting episode;<br>meeting response criteria after 10 weeks of open-label treatment with duloxetine (HAMD-17 score of 9 or less, CGI-S score of 2 or less, did not meet DSM-IV criteria for a major depressive episode);<br>provided written informed consent.
Exclusion criteria	Lifetime history of any psychotic disorder, bipolar mood disorder, or substance abuse disorder in the preceding year;<br>current or recent anxiety disorder that was a primary focus of treatment;<br>previous non-response to an adequate course of fluoxetine antidepressant treatment;<br>current episode unresponsive to 2 or more adequate courses of antidepressant therapy;<br>pregnancy or lactation;<br>patients with unstable medical conditions.	Type I bipolar disorder	Current and primary Axis I disorder other than MDD;<br>anxiety disorder as a primary diagnosis within 1 year of entry to the study;<br>treatment-resistant depression;<br>serious suicidal risk;<br>serious medical illness.	Current and primary Axis I disorder other than MDD, including but not limited to dysthymia;<br>a previous diagnosis of bipolar disorder, schizophrenia, or other psychotic disorders;<br>any anxiety disorder as a primary diagnosis within the past year;<br>an Axis II disorder that would interfere with compliance with the study protocol;<br>a DSM-IV defined history of substance abuse or dependence within the past year, excluding nicotine and caffeine;<br>a positive urine drug screen for any substances of abuse;<br>taking any excluded medications;<br>treatment with a monoamine oxidase inhibitor within 14 days prior to study entry;<br>treatment with fluoxetine within 30 days prior to study onset;<br>prior treatment history with duloxetine;<br>serious suicide risk;<br>serious medical illness likely to require hospitalization and/or the use of prohibited medications;<br>breastfeeding or pregnant women.

Table S2. Time points of Hamilton Depression Rating Scores from different protocols used in statistical analysis (in weeks after randomization to discontinuation treatment).

**Protocol**	**Baseline**	**Time 1**	**Time 2**	**Time 3**	**Time 4**	**Time 5**	**Time 6**
HCEX	Week 0	Week 2	Week 4	Week 10	Week 16	Week 22	Week 26
HCIZ	Week 0	Week 2	Week 4	Week 10	Week 16	Week 22	Week 25
HMBC	Week 0	Week 2	Week 4	Week 10	Average of week 14 and 18	Week 22	Week 26
HMDI	Week 0	Week 2	Week 4	Average of week 8 and 12	Week 16	Week 20	Week 24

Table S3: Estimated proportions by latent class and protocol in separate analyses of the four different protocols.

	HCIZ	HCEX	HMBC	HMDI	Overall
Low trajectory	12.0%	9.1%	17.1%	59.0%	44.3%
Middle trajectory	43.4%	52%	38.2%	10.7%	18.9%
Relapse trajectory	44.6%	38.9%	44.7%	30.3%	36.9%

Table S4: Estimated odds ratios for following the relapse trajectory in models with treatment as a predictor of trajectory membership.

	HCIZ	HCEX	HMBC	HMDI	Overall
Odds ratio for Active vs. Placebo, Low vs. Relapse	2.18 (0.92, 5.18)	3.64 (1.51, 8.76)	3.55 (1.43, 8.81)	2.07 (1.05, 4.06)	1.96 (1.37, 2.80)
Odds ratio for Active vs. Placebo, Middle vs. Relapse	1.47 (0.90, 2.41)	3.93 (1.94, 7.94)	2.64 (1.40, 4.96)	2.02 (0.77, 5.30)	1.98 (1.50, 2.62)

Figure S1. Study design of open-label and double-blind continuation phases of Protocol HCIZ.

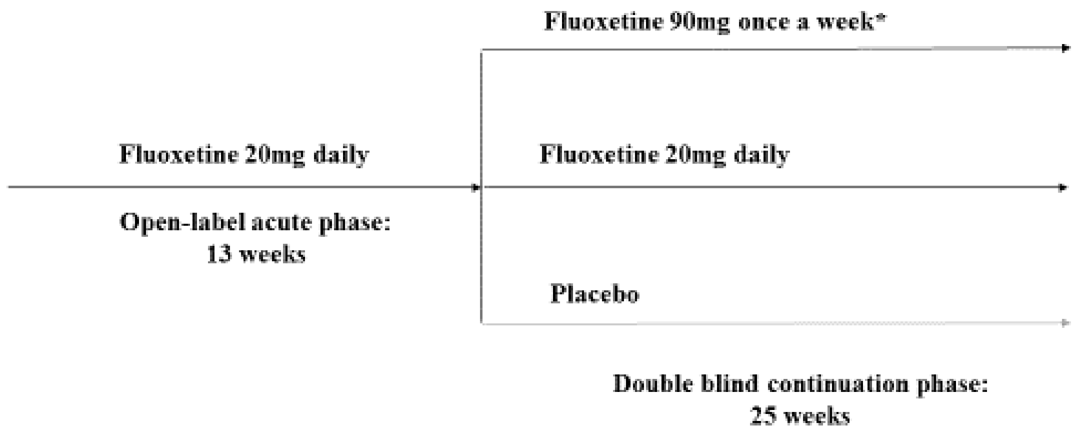

***Enteric-coated fluoxetine 90mg once a week with matching placebo on the remaining days of the week.**

Figure S2. Study design of open-label and double-blind continuation phases of Protocol HCEX.

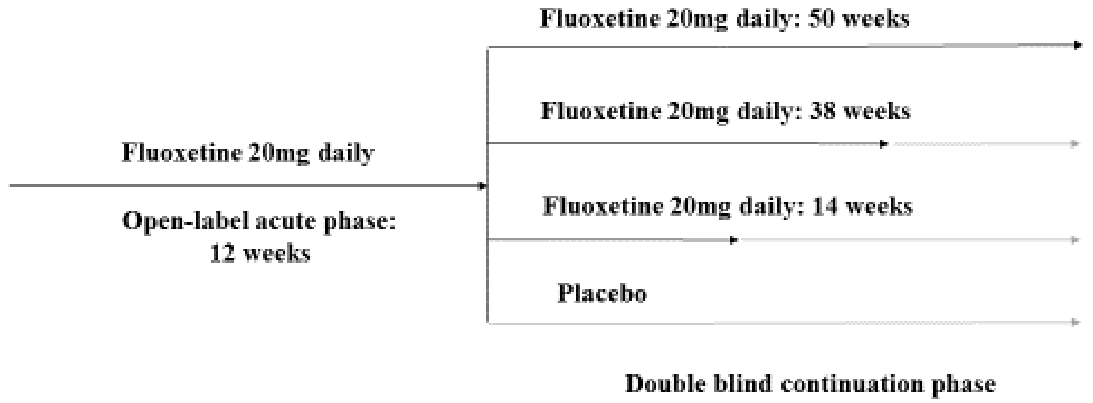

Figure S3. Study design of open-label and double-blind continuation phases of Protocol HMBC.

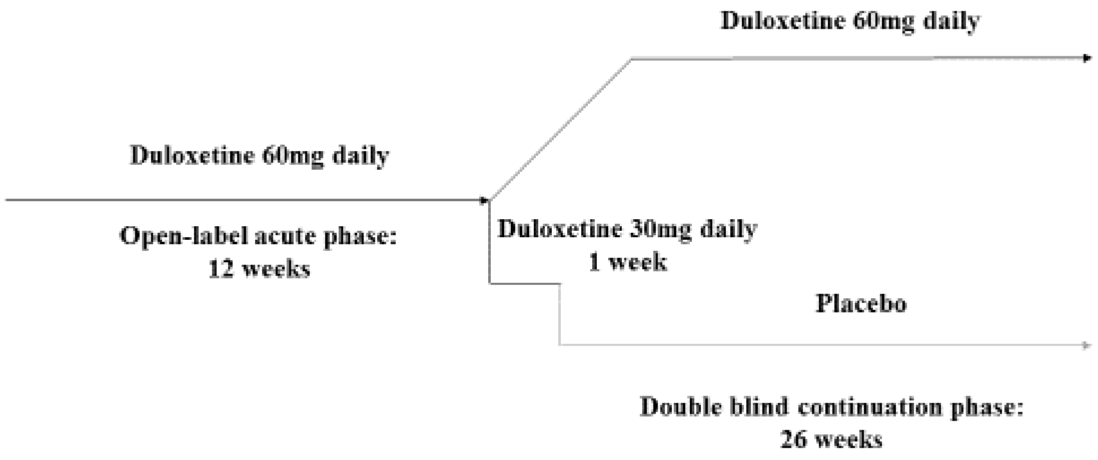

Figure S4. Study design of open-label and double-blind continuation phases of Protocol HMDI.

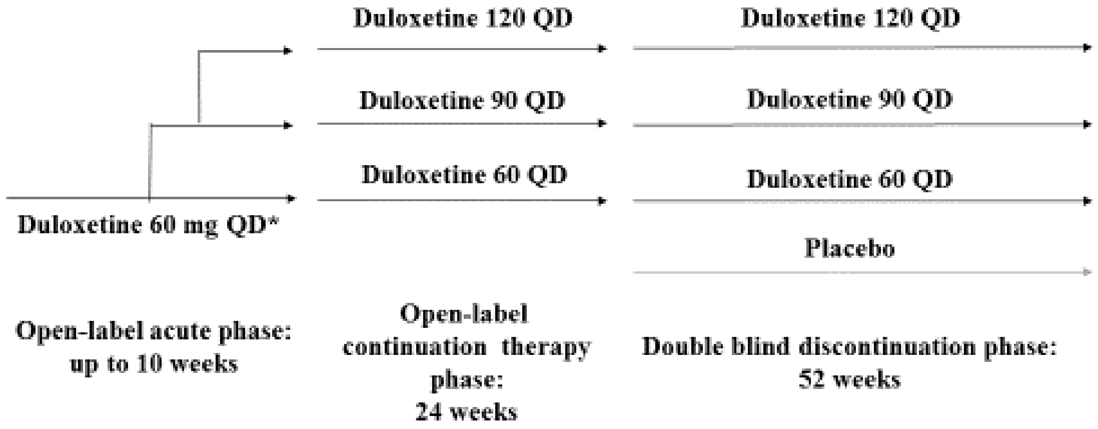

QD: once daily

*Patients who can't tolerate the 60mg QD dose can decrease to 30mg during weeks 1 to 3.
Patients who do not respond to 60mg can increase their dose to 90mg QD or 120mg QD.
Patients who meet response criteria between week 4 and 10 move directly to the open label continuation therapy phase.

Figure S5: Trajectories of HAMD scores during discontinuation in the HCIZ study.

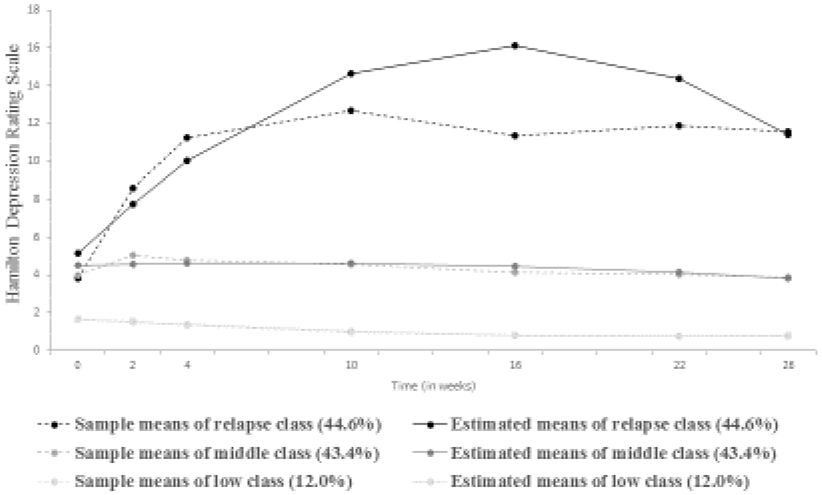

Figure S6: Trajectories of HAMD scores during discontinuation in the HCEX study.

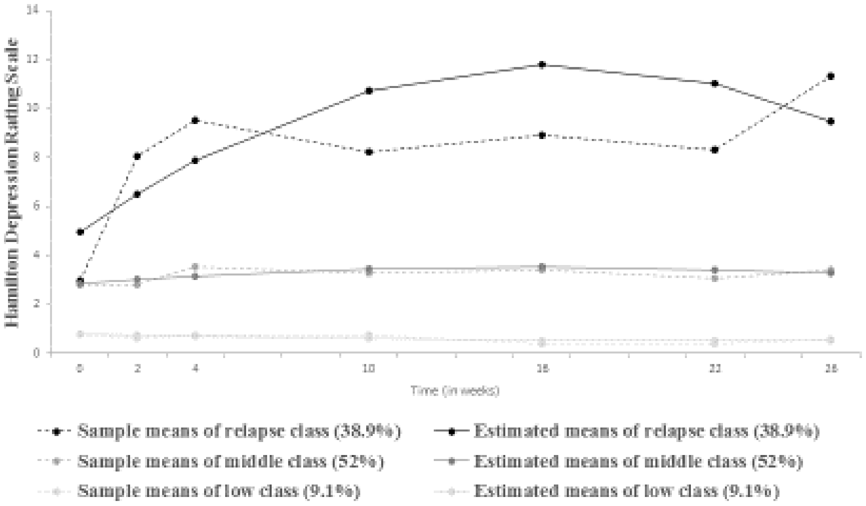

Figure S7: Trajectories of HAMD scores during discontinuation in the HMBC study.

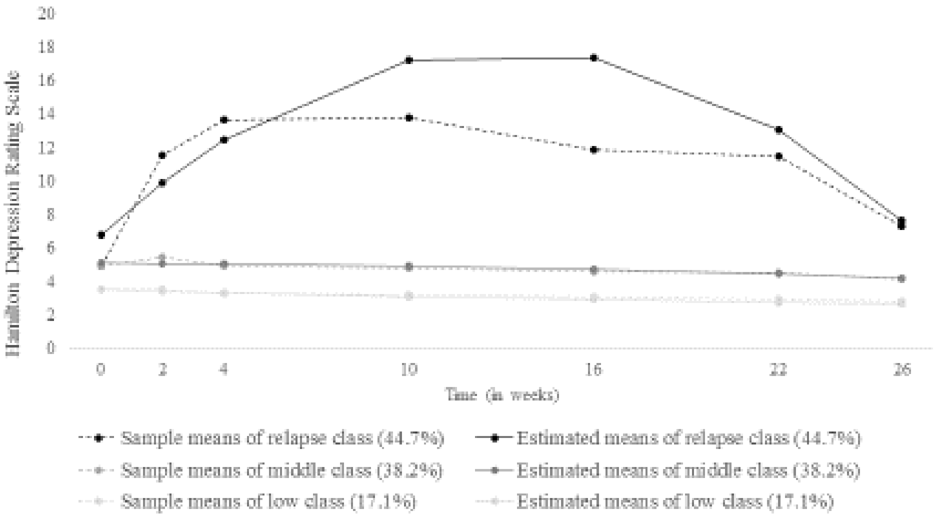

Figure S8: Trajectories of HAMD scores during discontinuation in the HMDI study.

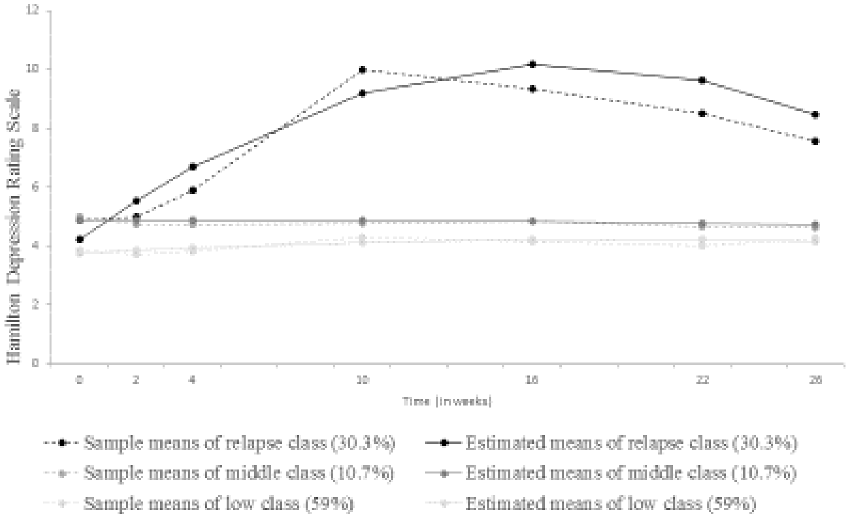

Figure S9: Observed Hamilton Depression Rating Scores of individuals most likely to be classified in each trajectory class with estimated mean trajectory superimposed.

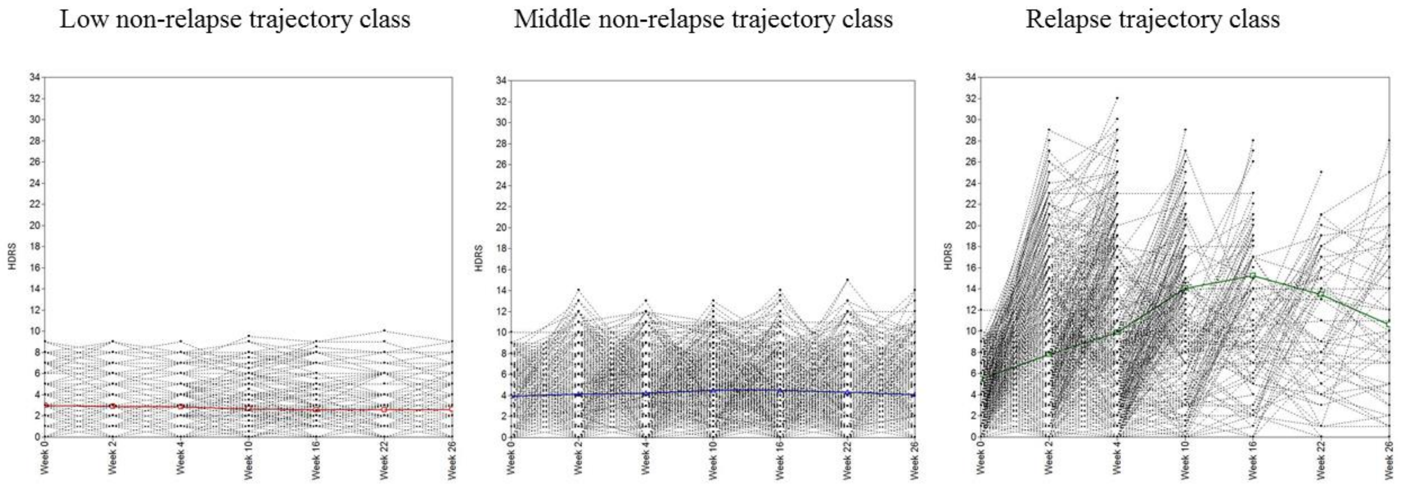

Figure S10: Mean Hamilton Depression Rating Scale scores over time for individuals classified as relapsers by both trajectory and clinical definition (Relapsers) and individuals classified as trajectory relapsers but not meating clinical criteria (Contested).

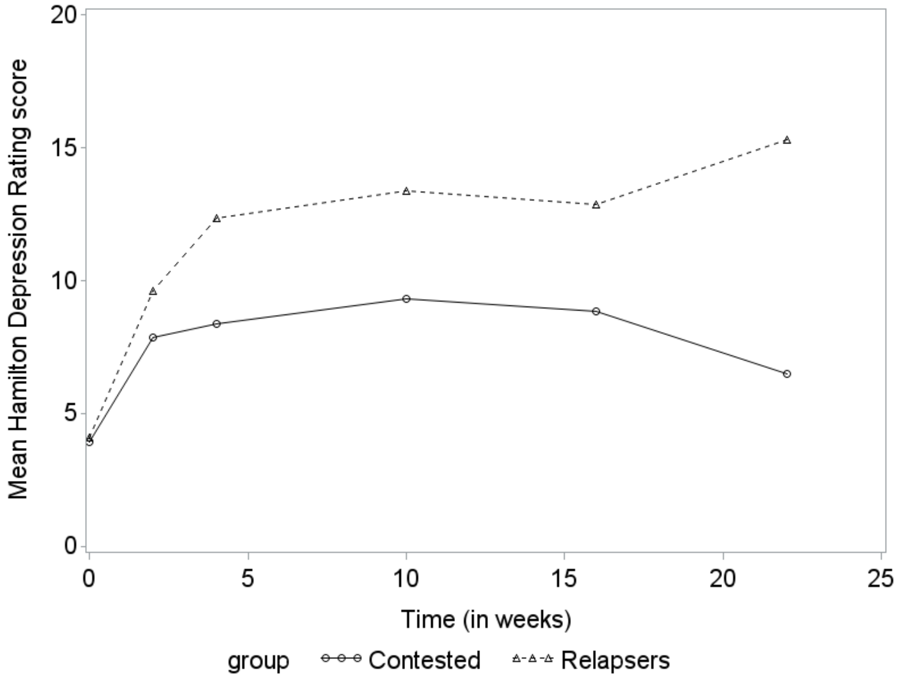

Figure S11: Receiver Operator Characteristic (ROC) curve for prediction of relapse trajectory from baseline predictors using weighted logistic regression model.

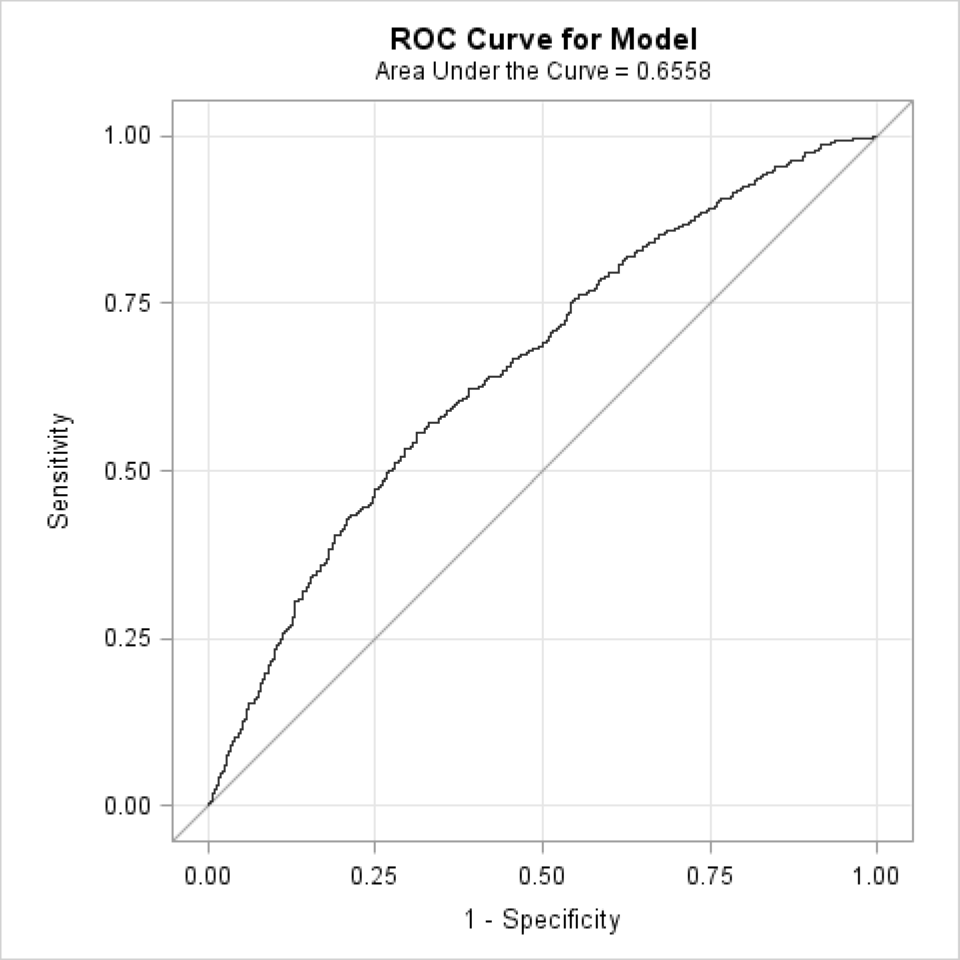

AUC = 66%

Balanced accuracy [(sensitivity + specificity)/2] at optimal cutoff = 60.6%
